# Neuroanatomical correlates of musical transposition in adolescents: a longitudinal approach

**DOI:** 10.3389/fnsys.2013.00113

**Published:** 2013-12-17

**Authors:** Mary Elizabeth Sutherland, Tomáš Paus, Robert J. Zatorre

**Affiliations:** ^1^Department of Neuropsychology, Montreal Neurological Institute, McGill UniversityMontreal, QC, Canada; ^2^BRAMS LaboratoryMontreal, QC, Canada; ^3^Rotman Research Institute, University of TorontoToronto, ON, Canada

**Keywords:** musical abilities, mental transformations, intraparietal sulcus, fMRI, development, adolescent

## Abstract

Musicians are trained in melodic transposition, the skill of extracting the pitch interval structure (i.e., the frequency ratios between pitches) and moving it into different keys (i.e., different pitch levels). This ability to recognize whether a melody is the same or altered when it is played back in a different key is correlated with both greater neural activation and cortical thickness in bilateral intraparietal sulcus (IPS). Musical training only explains part of this finding, suggesting that the ability to transpose a melody may have innate predispositions. The current study was designed to address this question: are the anatomical correlates of musical transposition already present in non-musician children at 14 years of age? If so, is there any evidence that those traits were already in place at earlier ages? To answer this question, we recruited 47 adolescents (age 14.5 years) from a longitudinal study and tested them on a melodic transposition task. These adolescents had already undergone anatomical magnetic resonance imaging (MRI) at the ages of 10 (Time 1), 11.5 (Time 2), 13 (Time 3) years, as well as at age 14.5 years (Time 4) They were tested on the transposition task during Time-4 visit. During this visit, we found a relationship between cortical thickness in left IPS and performance on the transposed melody task in the girls and not the boys; no such relationship was observed at any of the earlier ages. Given that girls reach more advanced staged of pubertal maturation earlier than boys, it is possible that the relationship between cortical thickness in IPS and skill at melodic transposition only emerges once the brain has reached a certain degree of maturity. This claim is supported by a lack of similar sex differences in the adults: the degree of correlation between cortical thickness and performance on the same transposed melody task did not differ between men and women in a previous study. Taken together, our results suggest that the relationship between cortical thickness and the ability to transpose a melody is not fixed, and that the effects observed in adults are neither due exclusively to training nor to predisposition.

## Introduction

Musicians have spent years developing a very specific set of skills in auditory, motor and cognitive domains, which are reflected to some extent in the structural brain differences between them and their non-musician counterparts (Schlaug et al., 1995a,b; Gaser and Schlaug, 2003; Bermudez et al., 2009). One of these skills is melodic transposition, which occurs when a certain melody retains its pitch interval structure (i.e., the frequency ratios between pitches stay the same) but the pitches themselves are changed. This ability to recognize intervals—regardless of the absolute pitches—is a fundamental skill for all musicians and therefore is frequently taught in any type of music classes. Repeatedly practicing interval recognition, both when intervals are presented in isolation and when they are presented as part of a melodic line has an effect: musicians tend to outperform non-musicians in such musical tasks (Trainor et al., 1999; Fujioka et al., 2004; Schon et al., 2004; Foster and Zatorre, 2010b). While this is generally the case, there is also a subset of people who have not undergone explicit musical training but who are able to perform such tasks, sometimes as well as trained musicians (e.g., Foster and Zatorre, 2010a,b).

These behavioral findings have functional and structural neural correlates. A functional imaging study (Foster and Zatorre, 2010b) investigating this particular skill used a simple same/different task: Participants with varying degrees of musical training listened to two melodies, the second of which was either transposed to a different key, or presented in the same key in a control condition. This second melody was either an exact transposition, or maintained the contour but changed one of the intervals. Participants were asked to respond whether the two melodies were the same or different. A contrast of the transposed and same-key melody conditions showed functional magnetic resonance imaging (fMRI) response in bilateral intraparietal sulcus (IPS); the magnitude of the response predicted performance both in musicians and nonmusicians. A second study using a similar paradigm replicated these results, and showed that fMRI response in IPS is a function of the musical key distance between the pairs of melodies (Foster et al., 2013).

The IPS is a region implicated in object rotation (Jordan et al., 2001), arithmetic processing (Kong et al., 2005), and in auditory temporal-reversal tasks (Rudner et al., 2005; Zatorre et al., 2010). What is common to all of these functions of the IPS is that they require abstracting perceptual information and transforming it in some way. Of special relevance to the present study, these functional findings are also reflected in a structural imaging result: in another study using the same transposed melody task in adults, Foster and Zatorre (2010a) found that both cortical thickness and gray-matter density in bilateral IPS correlated positively with the performance on the transposed melody task, even after taking account of musical training. The implication is therefore that there is a structure-function relationship such that those individuals with particular anatomical organization in this area have an enhanced behavioral ability (or that frequent engagement in this behavior leaves a structural signature).

These previous studies on manipulating melodic information have only tested adults, raising the question of whether the effects observed arise during development, or were pre-existing; in other words, were the people who subsequently became musicians just born with thicker cortices in bilateral IPS or did their cortices become thicker due to experience-induced plasticity? If the only observation of a relationship between cortical thickness and performance occurred in musicians, it would make sense to assume that this structure-function relationship was due to musical training, as many studies have demonstrated structural brain differences between musicians and non-musicians (Munte et al., 2002; Bengtsson et al., 2005; Jancke, 2009; Wan and Schlaug, 2010; Herholz and Zatorre, 2012). The fact that non-musicians as a group show similar behavioral and neural correlates as musicians (Foster and Zatorre, 2010a,b) suggests, however, that musical training does not explain all of the relationship between brain structure and ability on the transposed melody task. Thus, it is possible that there are predispositions for this skill, though to date none have been shown. The current study investigates exactly that question: could there be a neural “signature”, with regards to neuro-anatomical characteristics displayed at an earlier age, which could predict the later relationship between IPS structure and melodic transposition ability?

In order to investigate this question, we tested adolescents with a mean age of 14.5 years on the transposed melody task used by Foster and Zatorre (2010a,b). This age was approximately 10.5 years younger than the mean age of participants tested by Foster and Zatorre (2010a) and was chosen because it was an age at which many cognitive functions are already adult-like (Luna et al., 2004) yet during which the brain is still developing (Paus, 2005). These adolescents were already part of a longitudinal study; they were scanned at 1.5 year intervals since the age of 10 years. We hypothesized that at their last visit (at age 14.5) they would show a similar relationship between cortical thickness in bilateral IPS and performance on the transposed melody task, as observed in the adults in Foster and Zatorre (2010a). The advantage of testing a longitudinal cohort was that we would be able to determine not only if this task can be performed by adolescents, but if so, whether the neuroanatomical findings of Foster and Zatorre (2010a) extend to these younger ages. In addition, if these 14.5 year olds can do the task and show similar neuro-anatomical markers, we would be able to investigate whether this skill (tested at 14.5) is predicted by such a structural signature measured up to 5 years earlier. At this point, the parietal cortex is still not yet mature (Giedd et al., 1999; Paus, 2005). We were also interested in seeing whether a potential relationship between cortical thickness in IPS and the transposed melody task was related to pubertal maturation. By the age of 14.5, girls tend to be more advanced than boys (Tanner and Whitehouse, 1976). Our null hypothesis was that this relationship between cortical thickness in the IPS and performance on the transposed melody task could be explained by predispositions, in other words, that children as young as age 10 should already show a similar effect. However, if these neuroanatomical markers develop later in life, we might expect a difference between boys and girls, as they would be at different stages of puberty by the later time points (Sutherland et al., 2012). This differing maturational trajectory is also reflected in the neural development. For example, males show peaks in gray-matter 1–2 years later than females (Lenroot et al., 2007), and also appear to have a steeper increase in the volume of white matter during adolescence (Perrin et al., 2009). This difference between the sexes should subsequently diminish or disappear as they reach corresponding levels of maturation.

## Materials and Methods

### Participants

A total of 65 healthy English-speaking 10-year old children were initially recruited for a longitudinal study of normal adolescent development, 47 of whom (21 male, 26 female) successfully completed the auditory component. The study involved four separate 2-day visits across 4.5 years during each of which brain imaging studies, psychometric assessment and behavioral testing were carried out. Children and parents also completed a number of questionnaires. Some data had to be excluded due to imaging artifacts (e.g., those caused by braces or head movement, incomplete data, or dropout), leaving complete imaging and behavioral data for 30 children (17 male, 13 female) at Time 1 (10 years old); for 43 children (24 male, 19 female) at Time 2, 18 months later; for all 47 children (21 male, 26 female) at Time 3, 36 months later; and finally for 43 children (24 male, 19 female) at Time 4, 54 months after the initial visit. All participants passed a neurological screening test before being enrolled in the study. Written informed consent (parents) and assent (children) was obtained in accordance with the ethical approval granted by the Montreal Neurological Institute Research Ethics Board.

All participants completed the Puberty Developmental Scale (Petersen et al., 1988), an eight-item self-report measure of physical development based on Tanner stages with separate forms for males and females at each of the four visits. There are five categories of pubertal status used in this scale: (1) pre-pubertal; (2) beginning pubertal; (3) mid-pubertal; (4) advanced pubertal; and (5) post-pubertal. Participants answer questions about their growth in stature and pubic hair, as well as menarche in females and voice changes in males. These self-report measures have been found to correlate with physician ratings of pubertal development (Dorn et al., 1990).

### Behavioral tasks

**Figure 1:**
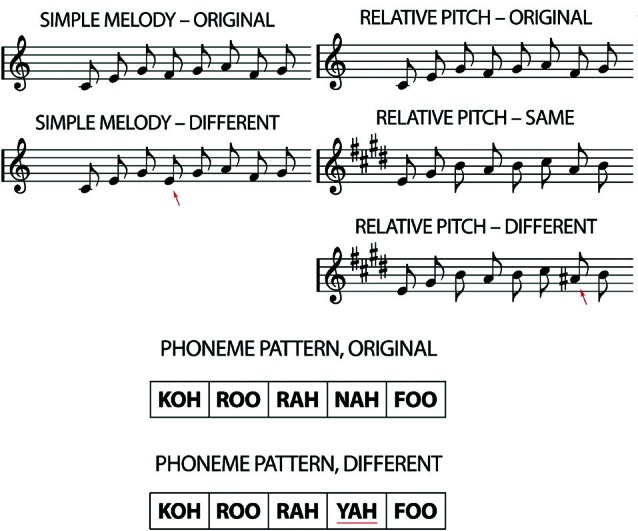
**Examples of the transposed melody task (relative pitch)**. The “original” played first, followed either by the “same” or “different”, where the red arrow indicates the alteration. The phoneme pattern “original” was either followed by the “original” again, or by the different. Again, the red underline marks the alteration (adapted from Foster and Zatorre, 2010a).

At Time 4, all children completed two same-different auditory pattern discrimination tasks (Foster and Zatorre, 2010a) a transposed melody as the main task of interest, and a phoneme task which served as a control (see Figure 1). Each trial consisted of two stimulus pattern presentations; participants judged whether the two patterns were the same or different by pressing either the left or right button of a computer mouse. Stimulus durations were varied within each task (i.e., the number of elements in each stimulus was varied), and the distribution of durations was matched among conditions. We chose to use varying durations for two reasons: (1) because this study is designed as a follow-up on those by Foster and Zatorre (2010a,b) and we therefore wanted to ensure compatibility with those data; and (2) because we were testing a heterogeneous sample of children not selected based on performance on this task, so we wanted to ensure that the task was sensitive to a range of musical experience. Participants filled out questionnaires regarding their musical experience, which included a detailed history of musical instruments played, which types of lessons they took (group, individual, self-taught), the ages at which they played, the musical environment in their house, and their general musical consumption (how much music they listened to, when, what genres). Thus, we were able not only to calculate the approximate number of hours played, but also to see whether they had spent a long amount of time seriously studying one or more instruments. These questionnaires were the same used by Foster and Zatorre (2010a,b).

### Transposed melody task

Stimuli in the transposed melody task consisted of unfamiliar melodies composed using the Western major scale, which ranged in length from 5 to 13 notes. The notes comprising the melodies were low-pass filtered harmonic tones and were between the pitches C4 and E6. All notes were 320 ms in duration, which is equivalent to eighth notes at a tempo of 93.75 beats per min. All the notes of the second stimulus pattern were transposed 4 semitones higher in pitch (in both the “same” and “different” trials). In the “different” trials, one note was altered by 1 semitone to a pitch outside the pattern’s new key, but one that maintained the melodic contour of the original melody.

### Phoneme task

The phoneme task was intended to control for auditory short-term memory. Stimuli were patterns of real speech consonant-vowel syllables (e.g., “ta”) spoken in a monotone. Like in the transposed melody task, patterns in the phoneme task ranged from 5 to 13 elements in length. The interval between phoneme onsets was 320 ms. The full set of phonemes consisted of 12 permutations of 8 consonants [b, k, f, n, p, r, s, f] and 4 vowel sounds [o, a, u, i]. The phonemes were selected to have minimal semantic association. In half the trials, one of the elements in the second pattern was changed to a different phoneme. The two stimuli in each trial always used different source recordings (of the same speaker) so that acoustical cues unrelated to phoneme identity could not be used as cues in making the same-different judgments.

### Image acquisition

Magnetic resonance imaging (MRI) data were collected using a Siemens 1.5-T superconducting magnet. High-resolution T1-weighted (T1-W) anatomical images were acquired using the following sequence: 3D RF-spoiled gradient echo scan with 140–160 slices, 1-mm isotropic resolution, TR = 22 ms, TE = 9 ms, flip angle = 30°. Participants were scanned with this protocol a total of four times: at the ages 10, 11.5, 13 and 14.5 years of age.

### Cortical thickness analysis

We used the CIVET pipeline (version 1.1.11^1^ (Zijdenbos et al., 2002; Kim et al., 2005)). This pipeline began by registering the T1 images to the ICBM152 nonlinear sixth generation template with a 12-parameter linear transformation (Collins et al., 1994; Grabner et al., 2006), RF inhomogeneity-corrected (Sled et al., 1998) and tissue-classified (Zijdenbos et al., 2002; Tohka et al., 2004). Subsequently, deformable models were used to create gray matter, white matter, and cerebrospinal fluid interfaces for each hemisphere separately (MacDonald et al., 2000; Kim et al., 2005). The result was four surfaces, with 40,962 vertices each. These surfaces were used to derive the *t*-Laplace metric using the Laplacian method for determining the distance between the white and gray matter surfaces (Jones et al., 2000; Lerch and Evans, 2005; Haidar and Soul, 2006). The thickness data were then blurred using a 20-mm surface-based diffusion blurring kernel in preparation for statistical analyses. Unnormalized, native-space thickness values were used in all analyses owing to the poor correlation between cortical thickness and brain volume (Sowell et al., 2007). Normalizing for global brain size when it has little pertinence to cortical thickness risks introducing noise and reducing power.

The degree of correlation between cortical thickness values and performance on the transposed melody task was determined across the whole cortical surface, using a vertex-wise general linear model analysis. This method estimates the covariation of cortical thickness with participants’ performance on the transposed melody task. An uncorrected threshold of *p* = .01 was used to determine which regions are potentially implicated in the relationship between cortical thickness and performance on the transposed melody task. We chose to use the uncorrected values to visualize subtle effects in neuroanatomy that we would expect during this period of adolescence, when the brain continues to mature. This state of change (e.g., Giedd et al., 1999; Sowell et al., 2001; Paus, 2005; Tamnes et al., 2010) could potentially decrease the signal to noise ratio and obscure more subtle findings. This study was predicated on a specific prediction based on earlier findings, and so we were interested in any correlation in the predicted area of the IPS.

## Results

### Transposed melody and phoneme task—Behavioral results

Our participants were non-musicians, and so showed a range from 41.6% to 81.7% correct (mean, 56.7% standard deviation 10.1%). As expected, they performed better on the phoneme task, where they achieved an average of 66.2% correct (standard deviation 8.45%). A one-way ANOVA demonstrated that there was no difference between boys and girls on either task; including puberty stage in the model also did not aid in explaining the task variance. There was also no correlation between performance on the transposed melody task and phoneme task (Pearson’s *r* = .085; *p* = n.s.), supporting our claim that the transposed melody task is measuring a specific musical skill and not just auditory short-term memory. We had also administered a questionnaire to determine the musical experience of our sample. We had no individuals who might be classed as “musicians” (people who were studying music seriously or had had more than 6 years of music lessons outside of the usual school curriculum); we calculated the total number of hours played (e.g., in garage bands, piano lessons at younger ages, group music courses etc.) and used these numbers in a Pearson correlation with performance on the transposed melody task. We failed to find a significant correlation. We also examined the total number of years spent playing a musical instrument (most often in music classes at school) and here also failed to find a significant Pearson’s correlation with performance on the transposed melody task.

### Transposed melody task—Cortical thickness

**Figure 2:**
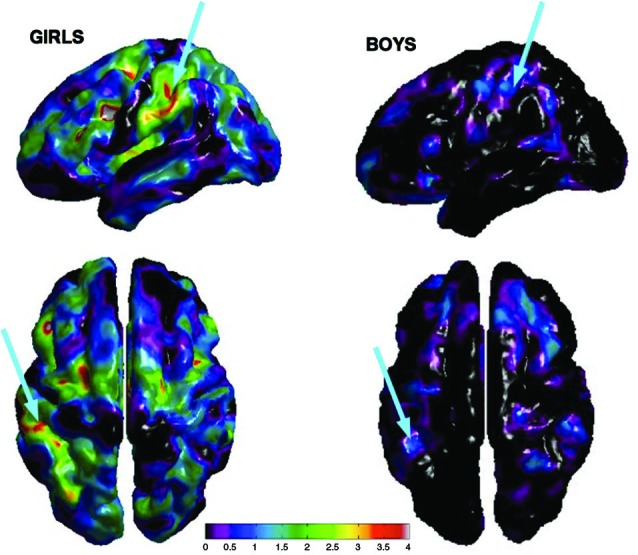
**Cortical thickness maps of bilateral IPS showing peak correlation between performance on the transposed melody task and CT at Time 4 for girls (left) and boys (right) separately**. Arrows show peak correlation in left IPS from both a lateral and dorsal angle.

**Figure 3:**
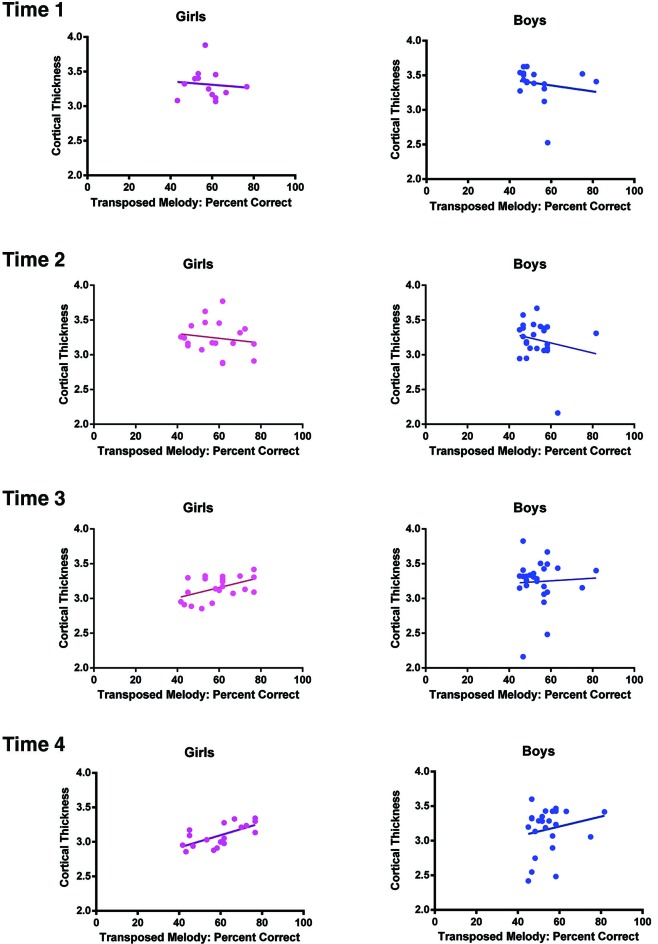
**Task performance on the transposed melody task plotted as a function of cortical thickness at the coordinates of the peak voxel in left IPS for girls (left) and the peak voxel in left IPS for boys (right) separately at each Time point.** At Time 1, the correlation between CT and task performance was *r* = −.096, *p* = .76 for girls, *r* = −.176, *p* = .5 for boys; At Time 2, the correlation was *r* = −.157, *p* = .52 for girls, *r* = −.195, *p* = .36 for boys; at Time 3, the correlation was *r* = .49, *p* = .02 for girls, *r* = .05, *p* = .81 for boys; and finally at Time 4, the correlation was *r* = .66, *p* = .002 for girls, *r* = .2, *p* = .35 for boys.

There was a significant difference in puberty level between boys and girls at all time points (Time 1, *p* < .001; Time 2, *p* < .001; Time 3, *p* = .002; Time 4, *p* = .012). Therefore, we separated our sample into males and females and analyzed the correlation between cortical thickness and performance on the transposed melody task separately. At Time 4, a full brain search showed a correlation between cortical thickness and behavior on the transposed melody task in the left IPS (*x* = −46, *y* = −29, *z* = 44), but only in the girls (*t* = 3.67, *p* < .002; uncorrected). The boys showed no corresponding correlation in left IPS, nor anywhere else in the brain (see Figures 2, 3).

**Figure 4:**
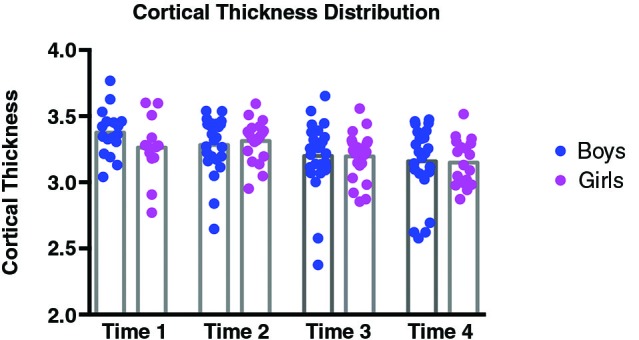
**Distributions of cortical thickness for girls (pink) and boys (blue) separately at each Time point**. At Time 1, average cortical thickness was 3.26 for girls, and 3.38 for boys; at Time 2, average cortical thickness was 3.31 for girls, and 3.28 for boys; at Time 3, average cortical thickness was 3.2 for girls, and 3.2 for boys; and finally at Time 4, average cortical thickness was 3.15 for girls, and 3.16 for boys. None of the differences in CT values between girls and boys were significant.

In order to examine directly the relation between the findings at Times 1 and 4 we selected a subsample of the girls for whom we had complete behavioral and imaging data at both time points. Because we had to discard some of the data from our sample (due to imaging artifacts, incomplete data, or dropout), the resulting sample size was 11. While the sample size was small, it did replicate the results of the larger group, with a correlation at the same location in left IPS with *t* = 3.76 (*p* = .005 uncorrected) at Time 4, but with no correlation in the left IPS at Time 1. It is important to note that there was neither an effect of sex nor puberty on the behavioral score on the transposed melody task; only a difference between the sexes in the strength of the correlation (*p* = .04, as determined using a Fisher *r*-*z* transformation). The cortical thickness in left IPS of both sexes showed a general decrease from Time 1 (average CT value 3.33) to Time 4 (average CT value 3.17), as is to be expected during adolescence (Sowell et al., 2001; Tamnes et al., 2010). There were no significant differences in the cortical thickness of left IPS between girls and boys (see Figure 3). In order to see how the measures of cortical thickness in left IPS changed with age, we computed the correlation between cortical thickness values for each of the participants between Time 1 and their cortical thickness at each subsequent visit. None of the correlations were significant (between Times 1 and 2, *r* = −.16, *p* = .38; between Times 1 and 3, *r* = .03, *p* = 86; between Times 1 and 4, *r* = .06, *p* = 71) which suggests that the cortical thickness of this area can change over this time period, such that its value at one time point is not predictive of the value at another time point (see Figure 4).

We then combined the girls and boys to see if the correlation between cortical thickness and behavior could still be seen in a whole-group analysis. As was expected, the *t*-value decreased (to 2.2; *p* = .03 uncorrected) and the location of the peak voxel shifted slightly (*x* = −40, *y* = −42, *z* = 48; 14.87 mm away from the peak shown in the sample of girls and 8.7 mm from the results obtained by Foster and Zatorre, 2010a). When we examined the data from the boys and girls separately at the location of the group peak, we found that the result was still driven primarily by the girls (*t* = 3.3) compared with the boys (*t* = .92). There were no other significant correlations between cortical thickness and performance on the transposed melody task anywhere in the brain.

In order to determine how this relationship between left IPS and performance on the transposed melody task developed over time in the whole group, we calculated the *t*-values at each Time point (age 10, 11.5, 13, and 14.5) at the location which was most significant at the oldest age. There were no significant correlations at any of the earlier time points: at Time 1 (age 10), the value was −.71, at Time 2 (age 11.5), the value was .93, at Time 3 (age 13), the value was 1.07 (see Figure 3). There was also no significant correlation between CT scores at the group peak and musical experience (measured either as years played or hours practiced, though those two were highly correlated).

In order to determine whether the higher correlation between the cortical thickness in left IPS and score on the transposed melody was due to the differing developmental trajectories of boys and girls and not sex differences per se, we re-analyzed the data from Foster and Zatorre (2010a) to examine the presence of any sex differences, which had not been reported in that study. We found no differences between males and females in the correlation between cortical thickness and transposed melody score at either the group peak in the left IPS reported in that study (*x* = −35, *y* = −46, *z* = 42; females: *t* = 3.1, males 3.56), or the coordinates of the peak voxel in the current study (females *t* = 1.8; males *t* = 2.1); rather, there were significant correlations in each of the two groups of adults.

### Phoneme task

The phoneme task was intended as a control task for the transposed melody task, to ensure that the transposed melody task was measuring the ability to manipulate auditory information, and not just auditory working memory or other nonspecific aspects of the task. A full-brain search of the data at Time 4 comparing performance to cortical thickness yielded no significant effects; the same was true at Time 1. As reported previously, there were significant differences in puberty between the boys and girls, so we repeated these analyses for the girls and boys separately, but no significant sex differences in the IPS emerged.

## Discussion

We found, as predicted, that cortical thickness in the IPS is correlated with behavioral performance on a melodic transposition task at age 14.5; however this effect was only seen in girls and not boys, and only observed on the left side. No such relationship was detected for a control phoneme task, also as predicted. When we tested for the existence of a relationship between behavior and anatomy at earlier ages we found no evidence of any significant effect at any prior age. Taken together, our results support the conclusion that a relationship exists between anatomical features in this part of the brain and a specific auditory ability, and that this relationship can be observed as early as at 14 years of age. However, the findings show no evidence to support the hypothesis that current performance on the transposed melody task is based on neuroanatomical precursors that are present at earlier ages. This set of findings could be taken as a disproof of the null hypothesis that the structure-function relationship is a stable and present since early in development, and this may indeed be the correct conclusion. However, we must exercise caution because the lack of a relationship at earlier ages could of course also be due to a number of other factors, such as insufficient sample size across time points, a measure too crude to pick up on a potentially more subtle precursor, too much variance in our sample, a task intended for adults and a bit too difficult for most of the adolescents we tested, or perhaps even to confounding variables outside our control (e.g., home or school environment). Thus, while we do find similar neuroanatomical correlates for the transposed melody task to our adult sample at least in the older girls, we cannot be sure that there are no precursors.

The IPS is far from adult-like at the age of 10 years (e.g., Giedd et al., 1999; Sowell et al., 2001; Gogtay et al., 2004; Paus, 2005). In fact, our data indicate a trajectory similar to other longitudinal data showing that the cortex is thinning with age during adolescence (Tamnes et al., 2010). However, there are intraregional and sex differences (Giedd et al., 1999; Paus et al., 1999) showing that cortical gray matter begins decreasing at the age of 10.2 in females, and only at 11.8 years for males (Giedd et al., 1999). These findings fit nicely with our maturational data, which show that the girls are significantly more mature than the boys, and thus may explain why it is only the girls who show a more adult-like relationship between CT in left IPS and the transposed melody task whereas the boys show no such relation. Had we been able to test the boys an older age, when their maturity levels were similar to the girls at age 14.5, we would expect a similar relationship between CT in IPS and performance on the transposed melody task.

Our results also demonstrate a lack of correlation between the cortical thickness at Time 1 and all other time points. This finding, taken together with the general decrease in the cortical gray matter of the IPS during adolescence, shows that CT values can vary considerably over the course of a year and a half (the time between data collection) for any given individual, while still maintaining a global trend. This is of great interest because it leads to a possible explanation for why we may not have found any effects at the earlier time points: The brain is changing markedly from one time point to another, in a way that varies between individuals, due perhaps to the influence of sex-linked maturational factors, and also possibly as a function of differential experience. Thus, a failure to find a neuroanatomical predictor of current behavior (i.e., 4.5 years after the initial Time 1 scans) could be explained by this variability. By the time both sexes reach adulthood, the difference we observed between male and female adolescents is no longer visible, as determined from a reanalysis of the earlier adult sample. While it is unclear why this difference between the sexes arises and why it later disappears, we think that it may have something to do with developmental stage, which in turn may be related to puberty: The correlation between cortical thickness and performance on the transposed melody task is only visible in the more mature girls, who are more adult-like. As this difference disappears with age, it seems likely that the difference between the sexes that we observe in our adolescent sample may be partially mediated by the different stages of maturation of boys and girls at age 14.5.

### Correspondence with previous research

Like Foster and Zatorre (2010a), we show that CT in IPS can predict performance on the transposed melody task. However, we only found significant results in left IPS, not bilateral IPS, and found nothing significant in the auditory cortex, whereas the prior study did (Foster and Zatorre, 2010a). That our findings were confined to the IPS could be due to the variance in the data: Our sample was quite heterogeneous not only in terms of auditory experience, but also in terms of maturation. It could be that this noise made it impossible to find a correlation in the auditory cortex, but maintained a relationship in the IPS, the region that is responsible for abstracting and manipulating information in not only the auditory (e.g., Zatorre et al., 2002; Foster and Zatorre, 2010a,b; Foster et al., 2013) but also in the visual (e.g., Jordan et al., 2001) modality. It would be interesting to look at skills in other modalities previously shown to involve the IPS (e.g., arithmetic; Kong et al., 2005) to see whether they show an adult-like relationship in 10 year olds, or whether they too seem to be a function of development. While this was out of the scope of the present study, we are encouraged to see that this retrospective method can provide insights into the relationship between neuroanatomical development and cognitive task performance.

While ability on the transposed melody task has been shown to correlate with musical training, it is not driven entirely by such formal practice (Foster and Zatorre, 2010a). Our study failed to find a correlation between musical training and performance on the transposed melody task, but that is likely due to the fact that none of the adolescents we tested were themselves studying music seriously (at the time of testing). In this way, it suggests that musical training is not the only experience-dependent variable that can alter the structure of the IPS, but that there may also be other variables that come into play. It was not in the scope of this study to test what those variables may be; however, our results do strongly imply that there are other factors that should be taken into consideration when looking at the emergence of the neuroanatomical correlates of musical skills. There are many complex cellular and other microstructural changes that lead to the measures observable using structural MRI (Zatorre et al., 2012), and these changes interact with development in complex ways. It will be important for future studies to disentangle which of these many possible anatomical features are most relevant for the types of structure-behavior correlations studied here.

## Conclusions

This study explores the possibility of predicting current behavior based on past neuroanatomical data. Such a method is potentially beneficial as it can answer one of the most easily-posed questions regarding a relationship between neuroanatomy and behavior: Maybe they were just born that way?! The results of the current study suggest that there may not be any neuroanatomical precursors for this particular structure-function relationship, at least in our sample of adolescent non-musicians. We also failed to find a relationship between performance on the transposed melody task and musical training. Previous results have shown a relationship with musical training, but that this training does not account for all the variance (Foster and Zatorre, 2010a), thereby suggesting that there are other factors involved. This suggests that musical training is only one of multiple factors contributing to the relationship between the cortical thickness in IPS and musical ability (as measured by the transposed melody task). While we are unsure as to what these factors may be we were able to show this relationship in our sample of non-musician female adolescents. Thus, while there is a developmental component to the relationship between cortical thickness and ability on the transposed melody task, it is not one that is mediated exclusively by formal musical training.

These finding do raise the question: How do these structure-function relationships emerge over time? Our results suggest that time and development play a key role in this process, but because our study was not designed to answer specific developmental questions we can only guess at answers. What we can conclude from this study is that it is possible to find adult-like structure—behavior relationships in the brain, and that these are not necessarily stable but rather change during adolescence.

## Conflict of interest statement

The authors declare that the research was conducted in the absence of any commercial or financial relationships that could be construed as a potential conflict of interest.

## Footnotes

^1^http://wiki.bic.mni.mcgill.ca/index.php/CIVET
